# How Prediction Based on Sequence Detection in the Cerebellum Led to the Origins of Stone Tools, Language, and Culture and, Thereby, to the Rise of Homo *sapiens*

**DOI:** 10.3389/fncel.2018.00408

**Published:** 2018-11-13

**Authors:** Larry Vandervert

**Affiliations:** American Nonlinear Systems, Spokane, WA, United States

**Keywords:** acheulean artifacts, cerebellum, cerebellar internal models, cerebellar sequence detection, language evolution, phonological loop, working memory

## Abstract

This article extends Leiner et al.'s watershed position that cerebellar mechanisms played prominent roles in the evolution of the manipulation and refinement of ideas and language. First it is shown how cerebellar mechanism of sequence-detection may lead to the foundational learning of a predictive working memory in the infant. Second, it is argued how this same cerebellar mechanism may have led to the adaptive selection toward the progressively predictive phonological loop in the evolution of working memory of pre-humans. Within these contexts, cerebellar sequence detection is then applied to an analysis of leading anthropologists Stout and Hecht's cerebral cortex-based explanation of the evolution of culture and language through the repetitious rigors of stone-tool knapping. It is argued that Stout and Hecht's focus on the roles of areas of the brain's cerebral cortex is seriously lacking, because it can be readily shown that cerebellar sequence detection importantly (perhaps predominantly) provides more fundamental explanations for the origins of culture and language. It is shown that the cerebellum does this in the following ways: (1) through prediction-enhancing silent speech in working memory, (2) through prediction in observational learning, and (3) through prediction leading to accuracy in stone-tool knapping. It is concluded, in agreement with Leiner et al. that the more recently proposed mechanism of cerebellar sequence-detection has played a prominent role in the evolution of culture, language, and stone-tool technology, the earmarks of Homo sapiens. It is further concluded that through these same mechanisms the cerebellum continues to play a prominent role in the relentless advancement of culture.

## Introduction

### A monumental neuroscience breakthrough

Some three decades ago, Leiner et al. (1986, 1989, 1991) proposed that just as the cerebellum contributes to the refinement and automaticity of motor skills, the cerebellum's connections to the prefrontal cortex and Broca's language areas (areas 44 and 45) contribute to planning and language. In essence, they proposed that, operating below the level of conscious awareness, the cerebellum contributed to increased levels of thought in the following way:

Cerebellar connections to Broca's area may not only increase the speed and skill of speaking but also confer other benefits on humans. Because Broca's area communicates with other association areas in the cerebral cortex, the cerebellar signals to Broca's area could increase the speed and skill of such intracortical communication. These communications between cortical association areas are said to comprise the language of thought (Luria, 1980). Therefore, the processes of rationale thought may be performed with increased speed and skill in the human brain as a consequence of its enlarged cerebro-cerebellar connections [notably during the last million years of human evolution] (1989, p. 1006).

In line with Leiner et al.'s foregoing proposal on the mechanism of thought, Ito (1997, 2008) described how through repetition not only does the cerebellum automate skills at their high levels but is behind both the phylogenetic and ontogenetic origins of thought in working memory.

The evidence that Leiner et al. (1986, 1989, 1991) provided for their foregoing breakthrough hypothesis that cerebellum assisted the association cortex in the skillful manipulation of thought was massive and solid. Accordingly, their watershed proposal spurred a huge amount of brain imaging research on the cerebellum's contributions to the motor, cognitive, and affective functions, and, specifically, the cerebellum's contributions to what they referred to as the “skillful manipulation of ideas” (1986, p. 444). Over the last three decades, Leiner et al.'s above—quoted proposal has been broadly confirmed and further extended (Ito, 1993, 1997; Akshoomoff et al., 1997; Desmond and Fiez, 1998; Dum and Strick, 2003; Strick et al., 2009; Balsters et al., 2010, 2013; Imamizu and Kawato, 2012; Marvel and Desmond, 2012; Stoodley et al., 2012; Bostan et al., 2013; Schmahmann, 2013; Leggio and Molinari, 2015; Moberget and Ivry, 2016; Vandervert, 2016, 2017a,b; Adamaszek et al., 2017).

Balsters et al. (2010, 2013) and Bostan et al. (2013) are of particularly strong support of Leiner et al. (1986, 1989, 1991) in finding that (1) cerebro-cerebellar connections between the prefrontal cortex and the lateral cerebellum have grown more in volume in recent evolution than the rest of the cerebro-cerebellar connections, and (2) that, in these two-way connections, the cerebellum contributes skill routines and strategies for both first—and second-order rule-governed information processing, the highest levels of idea manipulation.

Nearly a decade after (Leiner et al.'s, 1989) proposal that the cerebellum contributed to the process of rational thought, Akshoomoff et al. (1997) convincingly argued that the cerebellum contributes to the manipulation of such mental skills through the control of *attention*. The cerebellum's role in the control of attention in both motor and cognitive processes has been widely confirmed (e.g., Kellermann et al., 2012; Stoodley et al., 2012; Brissenden et al., 2015, 2017). Akshoomoff et al. proposed that attentional control (among other processes) is learned in the cerebellum in the following manner:

The cerebellum is a master computational system that adjusts responsiveness [toward optimization] in a variety of networks to obtain a prescribed goal [this would include the optimization of *attentional control* in the central executive in Baddeley (1992) working memory model] (Courchesne et al., 1994; Courchesne, 1995). These networks include those thought to be involved in declarative memory, working memory, attention, arousal, affect, language, speech, homeostasis, and sensory modulation as well as motor control. This may require the cerebellum to implement a succession of precisely timed and selected changes in the pattern or level of neural activity in these diverse networks. We hypothesized that the cerebellum does this by *encoding (“learning”) temporally ordered sequences* [italics added] of multi-dimensional information about external and internal events (effector, sensory, affective, mental, autonomic), and, as similar sequences of external and internal events unfold, they elicit a readout of the full sequence in advance of the real-time events [this readout is a *prediction*]. *This readout is sent to and alters, in advance [italics added], the state of each motor, sensory, autonomic, attentional, memory, or affective system which, according to the previous “learning” of this sequence, will soon be actively involved in the current real-time events* [italics added]. So, in contrast to conscious, longer time-scale anticipatory processes mediated by cerebral systems, output of the cerebellum provides moment-to-moment, unconscious, very short time-scale, anticipatory information (p. 592–593).

In cerebellum studies, of the above-described “encoding *(“learning”) temporally ordered sequences* [italics added] of multi-dimensional information about external and internal events” is referred to as the learning of cerebellar *internal models*^1^.

Akshoomoff et al. (1997) found the cerebellum to be important in *shifting* and *orienting* attention in patients with cerebellar damage and, in normal adults, they found the cerebellum to be important in *focusing* and *shifting* attention. Overall they concluded that, the cerebellum is important in optimizing the quality of sensory information necessary to the coordination of the direction of selective attention (shifting, distribution, orienting) leading to prediction and anticipation of future states of affairs.

### In the march toward *Homo sapiens*, the cerebellum fine-tuned the brain to a cause-and-effect world

Since, according the Akshoomoff et al. (1997), the cerebellum predicts sequences of future events which are then sent to (“alters in advance”), for example, working memory, by definition cerebellar internal models mirror *cause-and-effect relationships* pertaining to the brain's modeling of internal and external events. That is, our knowledge of cause-and-effect relationships is ultimately based not primarily on functions of the cerebral cortex, but on *simulations in the cerebellum* of those events as to how they fit progressively more refined states of goal attainment as conceived in working memory in the cerebral cortex.

It may seem that the brain's cerebral cortex would have its own, perhaps innate, knowledge of cause-and-effect relationships. However, Vandervert (2015, 2016, 2017a) has combined extensive cerebellum research with infant studies that, together, describe how beginning in infancy the cerebellum likely played the predominant role in establishing the foundation of cause-and-effect relationships via its computation of *sequence detection* (Akshoomoff et al., 1997; Leggio and Molinari, 2015) of objects and the body moving in space. He argued that this process provided the foundational cause-and-effect basis for the infant's visual-spatial working memory. As will be shown later in this article, this model of the cerebellar origin of cause-and-effect will become critically important to a new way to understand how the predictive and anticipatory roles of the cerebellum were predominant in the origins of stone-tool technology, language and culture and, thereby, the origins of *Homo sapiens*.

Strongly substantiating (Akshoomoff et al., 1997) foregoing cerebellar mechanism of sequence detection, Leggio and Molinari (2015) independently proposed that the “operational mode” of the cerebellum is sequence detection leading to prediction. It is well worth quoting Leggio and Molinari's sequence detection position, as it strongly reinforces the role of cerebellar *internal models* to specifically include the prediction of future events via higher-order cognitive processes (working memory):

According to this hypothesis, the cerebellum detects and *simulates* [italics added] repetitive patterns of temporally or spatially structured events, regardless of whether they constitute sensory consequences of one's actions in motor planning, expected sensory stimuli in perceptual prediction, or inferences of higher-order processes [e.g., *cognitive elaboration* [italics added] or social cognition]. The simulation allows internal models [in the cerebellum] to be created that can be used to make predictions about *future events* (italics added) that involve any component, such as the *body, other persons, and the environment* [italics added] (p. 36).

### Working memory

Working memory has been described by Baddeley (1992) as a multi-component “brain system that provides temporary storage and manipulation for complex cognitive tasks such as language comprehension, learning, and reasoning” [abstract]. Baddeley divided working memory into the following three subcomponents: (1) an attention-controlling system which serves as a “central executive,” (2) a visual-spatial sketchpad which manipulates visual images within an ongoing flow of visual-spatial experience, and (3) a phonological loop which both *stores* and *rehearses* speech-based information. In addition, Cowan (2014) defined working memory as, “the small amount of information that can be held in mind and used in the execution of cognitive tasks” (p. 197). In that same article Cowan also described working memory as *the cauldron of concept formation* and further argued that concepts are bound together through executive attention in working memory.

Within this overall framework of the components and conceptual formation in working memory, Leggio and Molinari (2015) cerebellar internal model simulations of the “cognitive elaboration” of higher-order cognitive processes indicates that the cerebellum refines working memory through sequence detection. This strongly supports Leiner et al. (1986) earlier proposal that the cerebellum contributes to the “skillful manipulation of ideas,” which itself is a reasonable definition of working memory. Vandervert (2015, 2016) suggested that Akshoomoff et al. (1997) and Leggio and Molinari (2015) complementary models of sequence detection place the cerebellum as the unconscious basis of prediction and anticipation in the origin and advancement of human socialization, language, and thought in working memory.

### Purpose

Within the foregoing framework of cerebellar sequence detection leading to prediction and anticipation of future states of affairs, it is the purpose of this article to make the case that the last million years of natural selection toward the three- to fourfold increase in the size of the cerebellum, especially its cognitive areas, was the key to the rise of *Homo sapiens*. It will be argued that the cerebellum played the *predominant* role in the rise of *Homo sapiens* via the following two homologically^2^ linked research traditions. *First*, the cerebellum-driven development of attentional control of cause-and-effect relationships and thus prediction in the working memory of the infant will be described. *Second*, it will be argued that increases in this same cerebellum-driven development of attentional control in working memory increased early human capacities for the mental and dexterous manipulation of these cause-and-effect relationships which undergirded the evolution of the advanced stone-tool technology and language of *Homo sapiens* (Vandervert, 2011, 2015, 2016, 2017b).

### The prominent role of the cerebellum in the foundational establishment of attentional control in the working memory of the infant

The most detailed behavioral research in which the infant's initial foundations of the central executive (attentional control) and conceptual processes of working memory can be clearly revealed is that of Mandler (1992a,b, 2008, 2010, 2012). Mandler's model of infant mental development is presented in this article's context of cerebellar prediction and anticipation, because to an astonishing degree it can be directly mapped onto (1) the unconscious learning of internal models in the cerebellum, and (2) the foundational development of attention-driven visual-spatial working memory that jibes with Baddeley (1992) model.

**Figure 1:**
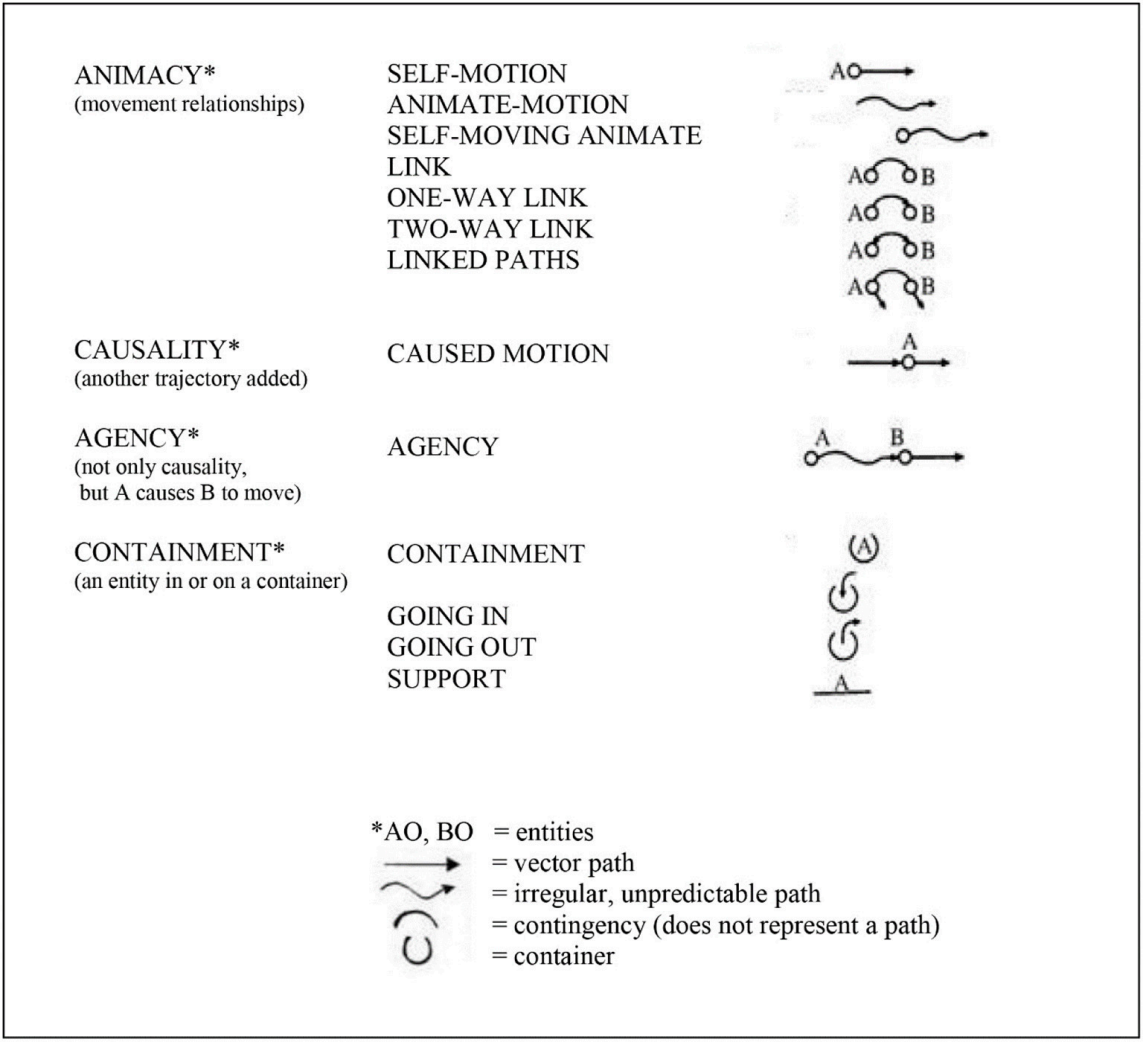
Mandler (1992a); Mandler's (1992b, 2008); Mandler (2012)) conceptual primitives—collectively, the infant's unconscious “primitive physics.” That is, the conceptual primitives represent the meaning of images and thereby establish the foundations of cause-and-effect relationships in the infant's visual-spatial working memory, notably the depicted categories of animacy, causality, and agency. It is argued that these conceptual primitives depict optimized predictive/anticipatory cerebellar internal models. It is not suggested that the conceptual primitives are constructed in the brain in this graphic form, but rather in the form of cerebellar microcomplexes as described by Ito (1997, 2008). Figure prepared by Kimberly Weathers-Moe Illustrations (kweathers10@mywhitworth.edu).

Mandler proposed that the infant repeatedly “notices” (pays attention to) specific aspects of its own bodily movement in relation to objects moving in the environment (the relationships among objects, space, and time), and that these movement parameters are “*distilled”* or “*condensed”* (1992a) into *conceptual primitives*. The infant uses these conceptual primitives to begin to understand and negotiate its environment (see Figure 1). (By “primitive” Mandler meant foundational, and did not mean unstructured, but structured).

Mandler (2012) proposed that this “distillation” process in the infant is the result of the infant's unconscious, *innately-initiated* and highly repetitive *perceptual meaning analysis* (PMA): “PMA is an attentional mechanism dedicated to simplifying spatiotemporal information. This innately driven “watching” on the part of the infant is activated by attention to objects, especially when they move, thus emphasizing the paths that objects take through space,” (p. 426). The infant's PMA doesn't simply “look” at the passing stimulus array, but repetitively “notices” and encodes aspects related to movement (Mandler, 1992b). Through this repetitive process of the noticing of movement and the distillation of the parameters of those movements, the infant derives the conceptual primitives consisting of animacy, causality, and agency (Figure 1). These conceptual primitives represent cause-and-effect relationships and thus permit the infant to predict and anticipate the effects of its own bodily movement in relation to objects moving in space.

Since the cerebellum encodes attentional (executive) patterns related to all repetitive motor, perceptual and working memory processes Ito (1997, 2008), Vandervert (2015, 2017a,b) proposed that these highly repetitive mechanisms of attention and distillation in Mandler's (1992b); Mandler (2012) perceptual meaning analysis in fact describe the process of cerebellar sequence detection toward *predictive* attentional control proposed earlier in this article by Akshoomoff et al. (1997) and Leggio and Molinari (2015). Since, within Vandervert's proposal, Mandler's conceptual primitives (Figure 1) are the result of cerebellar internal models of the attentional control of visual-spatial (spatiotemporal) information, they, within Baddeley's (1992) scheme, constitute the foundations of a *predictive* visual-spatial working memory. Accordingly, this means the infant's foundational visual-spatial working memory is adaptive because, as Akshoomoff et al. argue, *it is able to predict future states of affairs before they happen*. Indeed, in agreement with this position, Baddeley and Andrade (2000) argued that the key evolutionary advantage of bringing increasingly detailed memory into the current cognition in the form of working memory was/is prediction:

Baddeley (1993, 1998, chap. 18) has proposed that working memory plays a central role in the processes underlying consciousness, and that it has evolved as a means of allowing the organism to consider simultaneously a range of sources of information about the world, and uses these processes to set up mental models that facilitate the *prediction* [italics added] of events and the planning of action. Consider, for example, the task of a hunter-gatherer who recollects that as this time of year a tree bears fruit near a waterfall in potentially hostile territory. In order to reach the tree safely, he may need to use remembered spatial cues, together with the sound of the waterfall and the shape of the tree, while listening and looking for signs of potential enemies. A dynamic image that is capable of representing these varied sensory features simultaneously is likely to provide a planning aid of considerable evolutionary value (p. 128).

### Prediction intensifies with the evolution of working memory toward acquisition of the phonological loop

Within a pre-human scenario of stone-tool evolution, Vandervert (2011) offered a somewhat detailed explanation of how, as in the infant, cerebellar *sequence detection* in early Homo might have led to the *decomposition and re-organization* (Imamizu et al., 2007) of visual-spatial working memory *with* accompanying vocalizations and, thereby, more refined levels of prediction. He argued that this cerebellar decomposition and re-organization within working memory provided early Homo the powerful selective advantage of increased detail in observable cause-and-effect relationships pertaining not only to stone tools themselves but to social interactions related to stone-tool making and use. Thus, executive control (attentional control) in working memory gained the capacity to focus on more detailed cause-and-effect aspects of the physical and social environments. Vandervert argued that this cerebellar decomposition and re-organization of existing visual-spatial working memory with vocalizations provided the adaptive selection basis for the phonological loop of working memory, the second slave component of working memory proposed by Baddeley (1992). Vandervert further argued that this adaptive selection of the phonological loop parallels Mandler's (1992b, 2008) position that the infant's conceptual primitives (Figure 1) provide the bases for both simple inferential and analogical thought and the conceptual basis for the acquisition of the relational aspects of language. Via the acquisition of the adaptive phonological loop in working memory, then, more detailed cause-and-effect relationships could be mentally held and manipulated in working memory toward more refined predictions of future states, future states that became the framework for the adaptive origins of culture and, then, the relentless advance of culture (Vandervert, 2011, 2016). We will return to Vandervert (2011) pre-human stone-tool scenario below.

### Broader supportive evidence for the development of a visual-spatial working memory in the infant and a transition to phonological working memory in early childhood

In overall support of Vandervert's (2015, 2016, 2017a,b) interpretation of Mandler (1992b, 2008, 2012) infant studies, it is well established that the infant acquires appreciable visual-spatial working memory, especially between ages 6–12 months (Pelphrey et al., 2004; Reznick et al., 2004; Diamond et al., 2005). Moreover, there is strong evidence that, as Vandervert (2011) proposed, the cerebellum continues to play a predominant role in working memory development as the infant transitions from visual-spatial working memory to an added phonological component as the child develops. For example, Knickmeyer et al. (2008) argued that the 240% increase in the size of the cerebellum in the first year suggested that the cerebellum provides precisely the basis for such later development in the cerebral cortex:

Because the cerebellum is critically involved in motor coordination and balance (Bastian and Thach, 2002) the striking cerebellar growth may underpin the rapid motor developments of infancy. The cerebellum has also been implicated in a plethora of other cognitive abilities including planning, set-shifting, language abilities, abstract reasoning, *working memory* [italics added], and *visual-spatial organization* [italics added] (Schmahmann and Sherman, 1998). Given that “cognitive” regions of the cerebellum have reciprocal projections with non-primary frontal, parietal, and occipital association cortex (Thach, 1996), *the extremely rapid growth of the cerebellum in the first year may be a prerequisite for specific aspects of later cortical development* [italics added] (p. 12180).

Short et al. (2013) found significant associations between infants' (12 months of age) visual-spatial working memory and white matter tracts that connect brain regions known to support working memory in older children and adults. Within the context of Knickmeyer et al.'s (2008) above proposal that “the extremely rapid growth of the cerebellum in the first year may be a prerequisite for specific aspects of later cortical development,” Short et al. can be seen as strongly supporting the idea that the cerebellum may be a prerequisite for specific later cortical development [and thereby supported Vandervert (2011) proposal that the cerebellum continues to play a role in the transition to phonological working memory].

### The evolution of the cerebellum's dentate nucleus, a powerful clue to the cerebellum's important predictive role in working memory

Directly in this regard, it has been suggested that the evolutionary division of the cerebellum's dentate nucleus into motor and cognitive portions played a key role in the phylogenetic evolution of silent speech in within working memory (Marvel and Desmond, 2010a). The dentate nucleus of the cerebellum transmits learned movement and cognitive control models to the cerebral cortex (Leiner et al., 1986; Bostan et al., 2013). The dentate is divided into an older dorsal (motor) portion and a more newly evolved ventral portion (cognitive). Leiner et al. (1986) and Marvel and Desmond (2010a,b) provided strong evidence that the newer ventral dentate (cognitive output) was naturally selected *from* the evolutionarily older dorsal dentate (motor loop) as the cerebellar cortex and frontal areas of cerebral cortex expanded over the last million years. In humans, the cerebellum's ventral dentate is twice as large as the dorsal dentate and is proportionately larger than that of the great apes (Leiner et al., 1986; Bostan et al., 2013). The ventral dentate of the cerebellum outputs to the prefrontal, parietal and temporal areas of the cerebral cortex (Sokolov et al., 2017), and Marvel and Desmond (2010a) found that the dentate output served prefrontal and parietal language and executive working memory functions.

The cerebellum's dentate nucleus, then, appears to have evolved within the natural selection context of the adaptive advantage of a working memory able to quickly manipulate complex movement and mental skills in highly coordinated ways (Marvel and Desmond, 2010a). Based on extensive research studies, Bostan et al. (2013) argued that the “signal from the dentate to the prefrontal and posterior parietal areas of the cortex is as important to their function as the signal the nucleus sends to motor areas of the cerebral cortex” (p. 3). Thus, within the coordinated evolution of the cerebellum's dentate nucleus (from dorsal to dorsal-ventral) along with the parallel evolution of the prefrontal and parietal association areas of the cerebral cortex, the evidence strongly supports the idea that the rapid manipulation of mental skills evolved seamlessly within the context of the rapid manipulation of motor skills. It is suggested with Marvel and Desmond (2010a) that this certainly included the transition from the strongly motor-driven visual-spatial working memory (mostly dorsal dentate) toward the more cognitively driven phonological (silent speech-related) component of working memory (mostly ventral dentate) which would guide those motor systems in increasingly adaptive ways.

Thus Mandler's (1992b, 2008) idea that later, consciously accessible language concepts are built from the infant's visual-spatial conceptual primitives (Figure 1) and Vandervert's (2015, 2016, 2017a,b) interpretation that this sequence actually represents the foundations of working memory squares well with Knickmeyer et al. (2008) suggestion that the unparalleled growth of the cerebellum in infancy is a prerequisite for the later cognitive development of specific regions of the cerebral cortex. Likewise, in the next section of this article it will be argued that the unparalleled expansion of the cerebellum over the last million years was an undergirding requisite for cognitive developments of specific regions of the cerebral cortex related to a co-evolution of stone tool technologies and language.

### How language might have evolved from predictive decompositions and blends of visual-spatial working memory with vocalizations within the co-evolving context of tool use

Vandervert (2011, 2016) followed directly in the path of Mandler's (1992b, 2004; 2008) proposal that language develops/evolved from the foundational spatiotemporal primitive concepts shown in Figure 1. Vandervert argued that language was selected from vocalizations that were adaptively *blended* (Imamizu et al., 2007) with progressively more intricate visual-spatial image sequences required in the repetitive, structured sequences of tool use. Vandervert (2011) argued that in pre-humans and early humans, new environmental challenges set in motion the decomposition and re-organization of cerebellar internal models (Flanagan et al., 1999; Nakano et al., 2002; Imamizu et al., 2007; Imamizu and Kawato, 2009) of patterns of attentional focus on visual-spatial images and linked vocalization patterns related to stone-tool manipulation. These newly decomposed visual-spatial images and their linked sound patterns were re-organized or *blended* in the cerebral cortex (Imamizu et al., 2007) and error-corrected toward optimization in the cerebellum to meet the requirements of the new, challenging situation (Ito, 1997, 2008; Imamizu and Kawato, 2009).

**Figure 2:**
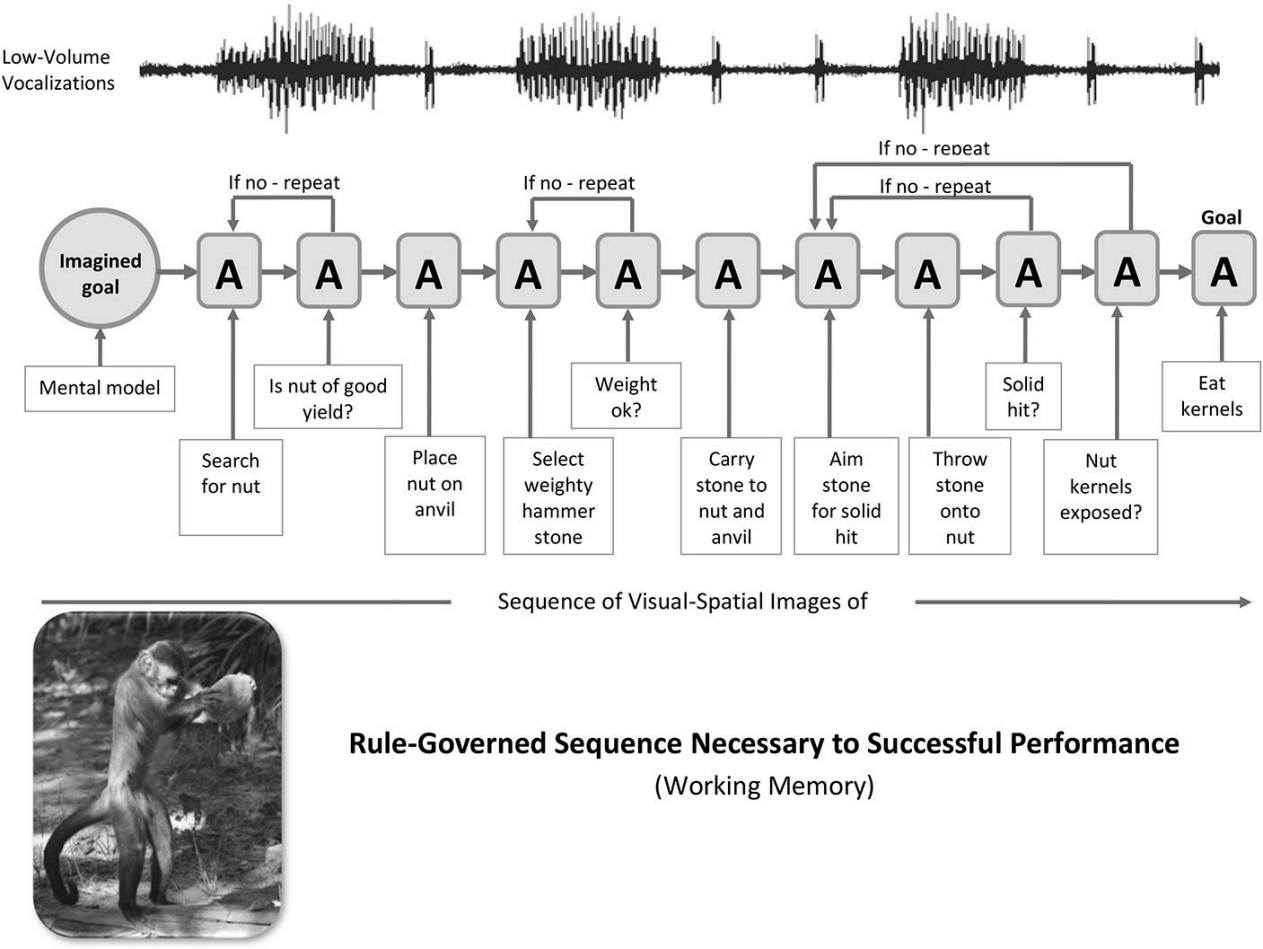
Capuchin Stone-Tool Use: A generalized sequence of stone-tool nut-cracking actions (A) driven by an imagined goal in visual-spatial working memory in capuchins. The stone-tool actions of the capuchin serve as a pre-human model for hand axe manufacture and use by early hominins between a million and a half and 150,000 years ago. Following the internal representation (imagined goal) of a prospective goal (I), the orderly series of actions (A) is mediated by cerebro-cerebellar loops. It is argued that the cerebro-cerebellar decomposition and blending of visual and vocal components of skill routines and strategies across the series of “If no—repeat” sub-routines of actions led to syntax (including the force component, i.e., verb forms) in working memory's phonological loop during language evolution. Photo of capuchin engaged in nut-cracking (lower left) obtained through Dr. Dorothy Fragaszy, Professor and Chair, Behavioral and Brain Sciences Program, University of Georgia and printed with permission of Barth W. Wright, EthoCebus Project (http://www.ip.usp.br/site/ethocebus). Figure prepared by Kimberly Weathers-Moe Illustrations (kweathers10@mywhitworth.edu).

A simple illustration of the cerebellar mechanisms of sequence detection, decomposition and blending that shows a movement-by-movement breakdown of the classic stone-tool sequences used by the pre-human example of capuchin monkeys was provided in Vandervert (2011). See Figure 2. This scenario draws directly from the findings of the extensive capuchin field research of Dorothy Fragaszy et al. (e.g., Fragaszy et al., 2013, 2017; Visalberghi et al., 2013; Mangalam et al., 2018). While the capuchin monkey is not in direct line with early Homo, several who have extensively studied, for example, the capuchin's (1) spontaneous stone tool selection, (2) bipedal stone transport, (3) highly refined manipulation of stone tools in nut-cracking, and (4) attention to the dynamics of stone hammer throw impact (Liu et al., 2011 have argued that the capuchin is an ideal model that can provide insights into the anthropological study of stone-tool use (Haslam et al., 2017. This is precisely the vein in which a cerebellum-focused analysis of the capuchin's use of stone tools is presented here.

In Figure 2, the capuchin's sequence of actions (A) begins with the internal mental representation (I) or goal involving stone-tool use on the left. The sequence of actions then progresses in a series of “if no—repeat” decisions in the capuchin's working memory. The resulting orderly action and decision-making defines a *syntax* of actions, as does syntax in speech. This syntax in capuchins is suggested to be the precise equivalent of the “abstract sequential operations” found to involve the collaboration of the cerebellum and the frontal and parietal areas of the cerebral cortex in monkeys (Obayashi et al., 2007). In Baddeley's (1992) model this mental processing in the capuchin would be that of visual-spatial working memory.

### Vocalizations paralleling the nut-cracking sequence: the earliest sub-vocal moments of the phonological loop

Dorothy Fragaszy, whose extensive research on capuchins is cited above, has indicated that capuchins in general “comment” on their manipulative activities with low-volume vocalizations (personal communication, 2011). Running across the top of the action sequence in Figure 2 is a line representing those ongoing low-volume vocalizations.

It is suggested that the vocalizations seen in Figure 2 accompany stone-tool use, because they serve a similar enhancing purpose for capuchins as does sub-vocal speech (silently talking to one's self) during intensive human tasks, especially those task intensively involving the hands. That is, it is hypothesized that sub-vocalization in capuchins helps to maintain cognitive focus on the immediate task at hand. This idea is supported by Marvel and Desmond (2010a) studies on how sub-vocal speech mechanisms enhance working memory in humans; they concluded as follows:

…the cerebellum enhances working memory by supporting inner speech mechanisms. This capability emerged from overt speech and motor systems as an evolutionarily adaptive way to boost cognitive processes that rely on working memory, such as language acquisition (p. 277).

In accordance with Akshoomoff et al. (1997) and Leggio and Molinari (2015) cerebellar sequence detection, the evolutionary adaptive advantage of low volume vocalization among capuchins would be that it enhanced attentional control in visual-spatial working memory that improved the prediction of successful goal attainment.

In summary here it is suggested that the cerebellar mechanisms discussed (decomposition and blending) are predominant in the processes shown in Figure 2. This predominant role of the cerebellum is supported by the foregoing evidence that the decomposition of vocalizations toward more articulated attention-sustaining sub-vocalization, and thereby enhanced prediction (Leggio and Molinari, 2015) would occur only through the learning of cerebellar internal models. These internal models would only then be sent to the cerebral cortex for possible blending with internal models of likewise decomposed visual-spatial images and thus the earliest evolutionary development of the phonological loop (Vandervert, 2011).

The foregoing scenario represents an emerging primitive physics as it may have occurred across generations of pre- and early humans. That is, the cerebro-cerebellar decomposition and blending processes would have resulted in the gradual emergence of a working memory where composites of images and vocalizations representing new cause-and-effect relationships could be quickly logged into long-term memory using sub-vocal or vocal tags. These phonological tags could subsequently be used to rapidly access the cognitive representations of cause-and-effect relationships from long-term memory to meet a variety of fast-moving environmental situations. The foregoing scenario provides an explanation for Baddeley et al.'s (1998) conclusion that the phonological loop selectively evolved due to its adaptive advantage of temporarily storing unfamiliar sound patterns while more permanent pathways were being constructed in long-term memory in the acquisition of language. The evolution of the phonological loop within the pre-existing visual-spatial working memory (and long-term memory) enabled the social sharing of detailed cause-and-effect relationships as well as the silent sub-vocal manipulation of ideas in planning, including the envisioning and manufacture of progressively advanced stone-tool technology.

### From capuchins to homo *Sapiens*

To illustrate this cerebellar decomposition and blending process in the evolution of greater prediction capacity of the phonological loop among Homo *sapiens*, I refer to leading anthropologists (Stout and Hecht, 2017) extensive research on how the cognitive, procedural and social complexities of stone-tool knapping, notably beginning with the complex, skill-intensive Late Acheulean period beginning 700 thousand years ago, might have led to the rise of cumulative culture. Stout and Hecht's robust research offers an ideal backdrop within which to illustrate many details of Leiner et al.'s (1986, 1989) last one million years of cerebellum evolution, including the cerebellum's prominent role in the accumulation of culture (Vandervert, 2016). Stout and Hecht's account of cultural evolution within stone-tool technology will be discussed in some detail, including the cerebellar mechanisms of both cultural origin and advancement as proposed by Vandervert (2016).

#### Repetitive complex skill-intensive stone-tool knapping: the last 700 thousand years

The central basis for Stout and Hecht's (2017) Acheulean procedural and social complexities argument for cumulative culture is seen is their following account of the rigorous technical and social requirements of stone-tool knapping:

Knapping is a “reductive” technology involving the sequential detachment of flakes from a stone core using precise ballistic strikes with a handheld hammer (typically stone, bone, or antler) to initiate *controlled and predictable* [italics added] fracture. This means that small errors in strike execution can have catastrophic, irreversible effects. Experiments by Bril et al. have shown that fracture prediction and control is a demanding perceptual-motor skill reliably expressed only in expert knappers (Greenfield, 1991; Haslam et al., 2017). Building on this work, Stout and et al. (Ito, 1993; Higuchi, 2007; Imamizu and Kawato, 2012) found that even 22 mo ($\bar{x}=$ 167 h) of knapping training produced relatively little evidence of perceptual-motor improvement, in contrast to clear gains in conceptual understanding (p. 7862).

In accordance with Akshoomoff et al. (1997) and Leggio and Molinari (2015) cited earlier, this highly precise, long-term, repetitive knapping would be mediated first and foremost through cerebellar sequence detection and error-correction toward attentional control and prediction, toward “controlled and predictable fracture.” Stout and Hecht (2017) continue directly on:

The key bottleneck in the social reproduction of knapping is thus the extended practice required to achieve perceptual-motor competence. This requires mastery of relationships, for example between the force and location of the strike and the morphology, positioning, and support of the core (Ito, 1997, 2007; Haslam et al., 2017), that are not perceptually available to naïve observers and cannot be directly communicated as semantic knowledge. Attempts to implement semantic knowledge of knapping strategies before perceptual motor skill development are ineffective at best (Ito, 2008, 2011), and such knowledge decays rapidly along knapping transmission chains when practice time is limited, even if explicit verbal teaching is allowed (Gallese, 2005). For *observational learning* [italics added], the challenge is to translate visual and auditory information of another's actions to appropriate motor commands for one's own body. This may be accomplished by linking the observed behavior with preexisting internal models [authors are here referring to models in the cerebral cortex, not in the cerebellum] of one's own body and actions through associative learning and stimulus generalization (Knickmeyer et al., 2008; Leggio and Molinari, 2015)….These learning challenges call for an interactive approach that alternates social-learning opportunities (observation, instruction) with motivated individual practice (Leiner et al., 1991), as commonly seen in coaching and apprenticeship practice (p. 7862–7863).

The first paragraph in the above quote describes the *repetitive*, fine *sequential* motor, and perceptual-cognitive requirements of stone-tool knapping, along with the integrally-related absolutely critical strike *prediction*/strike *error-correction* cycle. The second above quoted paragraph makes the key points of (1) “the challenge is to translate visual and auditory information of another's actions to appropriate motor commands for one's own body knapping mastery,” and (2) the therefore necessity of *social apprenticing* requirements for what the authors argue is necessary to the extreme rigors of complex knapping. Both of these points are well-reasoned and well-summarized, and it is agreed they are largely behind the evolutionary origin of culture and the subsequent ongoing evolution of culture. However, what brain mechanisms are actually involved in these processes, and how do they actually come about?

#### The more complete story behind the last million to 700,000 years of stone-tool evolution

Stout and Hecht (2017) placed the foregoing highly skilled stone-tool knapping processes exclusively in areas of the cerebral cortex, predominantly in the parietal, temporal and prefrontal areas. There is no doubt that these brain areas are importantly involved in the skilled tool knapping and manipulation description they provided. However, and this is a game-changing however, it is quite straight-forward to show that Stout and Hecht's account is seriously lacking as to the prominent contributions of mechanisms of the cerebellum in the following highly salient ways: (1) the cerebellum is involved in all complex movement and mental skill learning (Ito, 1997, 2008), (2) the cerebellum orchestrates the decomposition of and participates in the re-organization (blending) of these skills toward new skills (Baddeley, 1992; Nakano et al., 2002; Imamizu et al., 2007) (3) the cerebellum has been found to be involved in both the imagined and actual use of a variety of tools, with modular organization of internal models for imagined use of the various tools more lateral and posterior (Higuchi, 2007; Imamizu and Kawato, 2012). (4) the cerebellum automates imitative social learning of complex procedural spatial skills (Petrosini, 2007; Van Overwalle and Mariën, 2016), and (5) all of these cerebellar contributions occur below the level of conscious awareness. The addition of these five contributions of the cerebellum offer entirely new understandings of the predominately *unconscious* origin and accumulation of culture (Vandervert, 2016; Van Overwalle and Mariën, 2016).

Moreover, in a review of the cerebellum and non-motor functions, Strick et al. (2009) strongly supported this facilitative (and elaborative) role of cerebellar inner speech in working memory. In a fashion similar to that of vocalizations in the capuchin monkeys discussed earlier in this article (Figure 2), they suggested that the cerebellum is recruited whenever people engage in inner speech “to represent, maintain and organize task-relevant information and conscious thoughts” (p. 426), including in, for example, verbal working memory. It is proposed that as stone-tool knapping apprentices, in their early forms of language, overtly and silently “talked” themselves through the actual knapping process, this same inner speech process was adaptively selected across countless generations of cerebellar decomposition and the complex prediction requirements of “using precise ballistic strikes with a handheld hammer (typically stone, bone or antler) to initiate controlled and predictable fracture” (Stout and Hecht, 2017, p. 7862).

Overall, what Stout and Hecht have actually described in their above quoted social knapping arguments is an astonishingly congruent (and well-established) fit to (Adamaszek et al., 2017) the functions of the cerebellum (e.g., Bostan et al., 2013), and (Akshoomoff et al., 1997) it's evolutionarily concomitant volumetric anatomical and physiological changes which have occurred with stone-tool making over the last 700 thousand to one million years (Leiner et al., 1986, 1989). Thus, it can quite reasonably be shown that the actual brain mechanisms behind learning proficiency in stone-tool knapping would be dependent upon the procedural, cognitive and *socially contexted learning* of internal models in the cerebellum and their cerebellar error-correction toward proficiency optimization and innovation (Vandervert, 2016; Van Overwalle and Mariën, 2016).

Before moving on, it is important to emphasize that the foregoing cerebro-cerebellar approach does *not* necessarily conflict with (Stout and Hecht, 2017) proposed roles of the cerebral cortex in stone-tool manufacture. Rather, the cerebro-cerebellar approach brings to bear the functions of additional necessary brain mechanisms of the cerebellum that provide more detailed and more comprehensive explanations for how stone tool technology may have led to language and the origin of cumulative culture. The cerebro-cerebellar approach allows culture to be seen as a phenomenon that it was not in any way “thought out” by exceptional early humans or even as the product of the evolving “intelligence” of the cerebral cortex, but rather as the product of the silent, predictive role of the cerebellum constantly error-correcting toward optimization of complex motor, mental and social skills.

#### Predictive social learning contributions of the cerebellum

In their earlier quote in this article, Stout and Hecht (2017) made the strong point that for new learners, adequate stone-tool techniques “are not perceptually available to naïve observers and cannot be directly communicated as semantic knowledge” (p. 7862), and must be acquired through observational learning. Stout and Hecht further argued that therefore, “For *observational learning* [italics added], the challenge is to translate visual and auditory information of another's actions to appropriate motor commands for one's own body” (p. 7862).

In solid, preliminary support of such social learning contributions of the cerebellum, Van Overwalle and Mariën (2016) concluded that the cerebellum learns internal models for “social cognition” that are constantly error-corrected and sent to the cerebral cortex for the moment-to-moment, predictive “fluent and automatic social interaction” (p. 254). Van Overwalle and Mariën defined social cognition within the following context of observational learning: “Social cognition is the capacity to infer the social purpose of the behaviors of other persons or the self (i.e., “body” reading) and their state of mind (i.e., “mind” reading or mentalizing),” (2016, p. 248). This body- and mind-reading notion is in strong general agreement with the idea that the unique evolutionary expansion of human cognitive capacities was due to the demands of complex social interactions, as proposed in the *social brain hypothesis* (Dunbar, 1998, 2016), In this regard, Vandervert (2013, 2016) argued that, operating below the level of conscious awareness, the cerebellum learns and manipulates internal model *simulations* (Leggio and Molinari, 2015) of the actions and perceived thoughts of others, and thereby directly supported the social brain hypothesis as a product of the cognitive, emotional, and social functions of the cerebellum. Moreover, this cerebello-cerebral approach shows how social interactions became adaptively faster, more appropriate, and automatized, thus bonding social groups together and leading to cultural advances. The cerebello-cerebral approach strongly supports the *embodied simulation* explanations of the social brain hypothesis as espoused by Barrett and Henzi (2005) and Gallese (2005). At the same time, it is suggested that Vandervert's (2007) cerebellum approach is essential in clarifying the neural mechanism(s) behind this embodied simulation, and that it does this via internal models based on the sequence detection process articulated earlier in this article by Akshoomoff et al. (1997) and Leggio and Molinari (2015).

Due to the strong fit between Van Overwalle and Mariën's account of cerebellar social modeling, prediction, and error-correction and (Stout and Hecht, 2017) earlier quoted account of the rigors and social learning requirements of complex stone-tool knapping, Van Overwalle and Mariën's findings are presented in some detail as follows:

What is the function of the cerebellum in the service of social cognitive processes? The general assumption seems to be that an evolutionary older function of the cerebellum is to construct internal models of motor processes involving sequencing and planning of action, in order to automate and fine-tune voluntary motor processes. Scaffolding on this earlier function, a more recent function is to construct internal models [in the cerebellum] of purely mental processes during cognitive and social reasoning in which event sequences play a role (Ito, 2008; Pisotta and Molinari, 2014). This internal model is a copy [in the cerebellum] from the social event implications generated in mentalizing areas in the cerebrum (e.g., mPFC or TPJ), and allows humans to anticipate better action sequences during social interaction in an *automatic and intuitive way* [italics added] and to fine-tune these anticipations (p. 254).

Van Overwalle and Mariën's above cerebellum-driven capacity which “allows humans to anticipate [and predict] better action sequences during social interaction in an automatic and intuitive way” provides precisely the requirement for the social learning of stone-tool knapping outlined earlier by Stout and Hecht (2017). This strongly suggests that in the apprentice's observational learning in stone-tool knapping as described earlier by Stout and Hecht (2017), it is the cerebellum that provides the key neural mechanisms for sequence-detecting (e.g., Leggio and Molinari, 2015), error-correcting (e.g., Ito, 2008) and the automating (e.g., Hayter et al., 2007) of knapping toward optimal levels.

### A brief digression on working memory's relationship to automaticity

It may seem that automaticity learned in the cerebellum might lessen a person's online attention to or interfere with tasks at hand. However, along with automated sequences learned in the cerebellum, it has been shown that the learner maintains a complete online working memory focused on goals at hand (e.g., Hayter et al., 2007). In their above-quoted article, Van Overwalle and Mariën (2016) noted this necessity and indicated that their overall data provided evidence that the cerebellum was alerted whenever the automatic sequences it had learned were inappropriate to the situation at hand. Online working memory would then attend the specifics of such inappropriate matches and the cerebellum would respond accordingly.

Thus, the prominent role of the cerebellum in developing automaticity does not detract from or interfere with the ongoing, online conscious role of working memory. Rather, the cerebral cortex and the cerebellum constantly operate together to both consciously (in working memory) deal with immediate problems at hand while at the same time learning cerebellar internal models which, as Ito (1997, 2008) convincingly argued, unconsciously regulate the speed, consistency and appropriateness of these working memory operations. This allows automaticity to be both learned unconsciously and then to operate automatically below the level of conscious awareness (Leiner et al., 1986; Ito, 2011). This same back-and-forth between the cerebellum and working memory areas of the cerebral cortex would of course apply to an ongoing apprenticeship in the repetitive, skill-demanding stone-tool knapping described earlier by Stout and Hecht (2017).

## Conclusions and discussion

Some three decades ago Leiner et al. (1986, 1989, 1991) proposed that the cerebellum's 3–4-fold increase in the size and its projections to the parietal and prefrontal areas of the cerebral cortex in the last million years is an indication of its involvement in cognitive functions including language and the manipulation of thought. In this article it is concluded that while Leiner et al.'s proposal has been greatly extended by subsequent imaging research, it can now be even further extended to new levels of analysis via the mechanism of sequence detection (Akshoomoff et al., 1997; Leggio and Molinari, 2015). Understanding how cerebellar sequence detection further extends the cognitive functions of cerebellum provides more detailed explanations of the evolution of the interrelationships among stone-tool making, language and culture among Homo *sapiens*.

### Cerebellar sequence detection led to more detailed prediction of the outcomes of cause-and-effect relationships

Thus within the context of cerebellar sequence detection, further conclusions can be reached in at least three areas. *First*, as a result of its basic operation of sequence detection (Leggio and Molinari, 2015), the cerebellum can be seen to be the predominant player in the infant's foundational development of visual-spatial working memory. This cerebellum-driven working memory allows the infant's working memory to predict the movement of objects in relation to those of its body. *Second*, within the context of its sequence detection, the cerebellum can be argued to decompose and re-organize visual-spatial working memory and vocalizations in the adaptive selection of the phonological loop of working memory (Vandervert, 2011). This cerebellar decomposition and re-organization was adaptive because during long evolution of the phonological loop they predicted more detailed cause-and-effect outcomes related of food procurement in pre-humans through Homo *sapiens. Third*, it is further concluded within this cerebellar sequence detection context, that even though leading anthropologists Stout and Hecht (2017) provided a robust evolutionary neuroscience analysis of the origins of culture and language, their exclusive focus on functions of areas of the cerebral cortex can be seen as seriously lacking due to its omission of neuroscience detail that can be provided by newer imaging research on cognitive and language contributions of the cerebellum. Specifically, it can readily be shown that prediction necessary to complex, detailed visual-spatial stone-tool knapping and its socially contexted observational learning can best be explained not by functions of cerebral cortex alone, but by cerebro-cerebellar mechanism, with highly refined prediction predominately cerebellum driven.

These prominent cerebellum-driven contributions to the evolution of the cognitive functions behind stone tool making, language and culture include at least the following: (1) the cerebellum is involved in all complex movement and mental skill learning (Ito, 1997, 2008), (2) the cerebellum orchestrates the decomposition (Nakano et al., 2002) and participates in the re-organization (blending) of these skills toward new, more refined skills (Imamizu et al., 2007), (3) the cerebellum has been found to be involved in both the imagined and actual use of a variety of tools, with modular organization of internal models for imagined use of the various tools more lateral and posterior, thus producing a brain-based proclivity toward not only language learning but also tool use. (Higuchi, 2007; Imamizu and Kawato, 2012), (4) the cerebellum automates imitative social learning of complex procedural spatial skills (Petrosini, 2007; Van Overwalle and Mariën, 2016), and (5) all of these cerebellar contributions are learned and implemented below the level of conscious awareness (Leiner et al., 1986; Hayter et al., 2007; Ito, 2011).

In collaboration with the cerebral cortex the foregoing decomposition and blending in cerebellar internal models may be thought of as an “unconscious mode of thought” that leads to constant innovation and creativity, the earmarks of Homo *sapiens* (Vandervert, 2007, 2015; Ito, 2008). The cerebello-cerebral system apparently does this through the refinement of cause-and-effect relationships which opens new manipulative and thought horizons in language and tool configurations that can, through resulting refined cerebello-cerebral-driven prediction, further adapt Homo *sapiens* to its environment. At the same time, by so doing, it could suggest ways to alter that environment in new ways that were not previously found in nature. It is suggested that these adaptive cerebello-cerebral processes in the genus Homo and particularly Homo *sapiens* were/are the source of what leading anthropologist Holloway (1981, 2008) refers to uniquely human *arbitrary* forms. Within at least the last 700 thousand years it is suggested, in agreement with Holloway (1981), that these arbitrary forms included, for example, the evolution of language and of Acheulean stone tools.

## Author contributions

The author confirms being the sole contributor of this work and has approved it for publication.

### Conflict of interest statement

The author declares that the research was conducted in the absence of any commercial or financial relationships that could be construed as a potential conflict of interest.

## Footnotes

^1^Ito (2008, Glossary) defines internal models as follows: “Internal model: a dummy [in the cerebellum] of a body part or a mental representation in the cerebral cortex. It is encoded in the neuronal circuitry of the cerebellum and mimics essential properties of a body part or a mental representation for control [of movement or thought].” In other words, cerebellar internal models are models of the *internal* world going on in the cerebral cortex. The cerebral cortex on the other hand forms models of the relationships of the body to the *external* world and mental models of those relationships in, for example, working memory. Ito (2008) provides an excellent discussion of the roles of cerebellar internal models in a variety of contexts.

^2^The definition of *homology* here follows that of Greenfield (1991): In developmental psychology homology refers to common structural origins in the ontogeny of individual members of the species…. Whereas phylogenetic homology is defined as *descent from a common antecedent structure* [italics added] within an ancestral species, ontogenetic homology can be defined as descent from a common antecedent structure within the same organism (p. 533).
